# Reply to Matters Arising: Is congenital anosmia protective for Parkinson’s disease triggered by pathogenic entrance through the nose?

**DOI:** 10.1038/s41531-023-00539-4

**Published:** 2023-06-19

**Authors:** Artin Arshamian, Behzad Iravani, Johan N. Lundström

**Affiliations:** 1 Department of Clinical Neuroscience, Karolinska Institutet, Stockholm, Sweden; 2 Department of Neurology and Neurological Science, Stanford University, Stanford, CA USA; 3 Monell Chemical Senses Center, Philadelphia, PA USA

**Keywords:** Parkinson's disease, Olfactory system

**replying to** A. W. Fjaeldstad *npj Parkinson’s Disease* 10.1038/s41531-023-00538-5 (2023)

We have read with interest the “Matters Arising” commentary authored by Alexander Wieck Fjaeldstad, in which he comment on certain aspects of our recent article^1^. First, Dr. Fjaeldstad states that our title, which suggests that congenital anosmia could protect against the development of Parkinson’s disease (PD), does not align with the main hypothesis presented in the article. However, we respectfully disagree with this characterization given that our main hypothesis, as stated in the article, is that the absence of the olfactory bulb (OB) in individuals with isolated congenital anosmia (ICA) may serve as a protective factor against the initiation of PD in the OB. This hypothesis is supported by the proposed role of the OB as an entry point for pathogens or environmental components^2^. We explicitly state in the article, “Here, we propose a novel hypothesis that individuals with ICA might be immune to PD given the absence of their OB.” It bears emphasizing that not all cases of PD demonstrate a diminished sense of smell, as individuals with atypical parkinsonism, like multiple system atrophy, progressive supranuclear palsy, or corticobasal syndrome, typically do not display this symptom^3^. It is likely that the OB is not initially affected in these subtypes. This crucial differentiation is already mentioned in our original text, but merits further accentuation.

Our second hypothesis posits that the discovery of an individual with ICA, born without OB and subsequently developing PD, would contradict the hypothesis that PD onset occurs within the OB. As Dr. Fjaeldstad rightly points out, such a case would falsify the “Dual-Hit” theory, as it would eliminate one of the two proposed entry points^4^. However, our hypothesis is predicated on the assumption that the “Dual-Hit” theory is incorrect, and do not concern patients with body-first Lewy pathology. We should have stated this more explicitly and appreciate the opportunity to clarify this point. Our research interest is specifically focused on PD patients who display predominant pathology in connected olfactory structures (i.e., brain-first).

Recent findings by Borghammer and colleagues^5^ have provided compelling evidence that the pathological process of α-synuclein aggregation primarily initiates in the OB or enteric nervous system (ENS) plexuses, as well as the dorsal motor nucleus of the vagus, rather than simultaneously in the OB and gut. Of interest for our case is the notion that the OB might be an entry point for neurotropic pathogen that leads to α-synuclein pathology in the OB followed by secondary spreading to connected structures, such as the amygdala. It is in this scenario that our black swan would thrive and display a possibility for predominant pathology to connected olfactory structures, without an OB. This would effectively falsify that the entry point via the OB is a necessary condition for this type of pathology.

It is important to note that finding our postulated black swan does not negate all protective effect of ICA against PD. We argue that similar to the protective effect of vagotomy, as highlighted by Dr. Fjaeldstad, the absence of OBs would be beneficial in preventing, or solving PD pathology. For example, in the case of body-first cases, it could eliminate seeding to the OB from within the CNS, and thus slowing down the pathological progression^5–7^. Dr. Fjaeldstad expresses concern that recent case studies, which have uncovered individuals without apparent OB, yet displaying normal olfactory function, may complicate the diagnosis of potential ICA-PD^8^. However, we are not worried that the existence of such individuals will impede the diagnosis of ICA, as the morphological changes associated with ICA are distinct. For instance, individuals with ICA exhibit two specific abnormalities that both are distinct from the individual identified by Weiss and colleagues^8^ in that they lack functional olfactory receptors in the olfactory epithelium^9^, and they show large gray matter differences in the medial orbital gyrus and posterior orbital sulcus^10,11^. These morphological features are not present in those without OBs yet retaining normal olfactory function. As such, there are several ways to establish an ICA-specific morphological fingerprint for diagnostic purposes. That said, we fully endorse Dr. Fjaeldstad statement that the involvement of a clinician with experience of diagnosing ICA is essential. Dr. Fjaeldstad further suggest that the examination of the potential protective effects of olfactory dysfunction in PD should extend beyond the study of ICA cases. The investigation should also include other, more general forms of olfactory dysfunction, such as syndromic anosmia and anosmia resulting from head trauma. We agree that this expanded approach would be interesting, and improve the overall understanding of the association between olfactory dysfunction and PD. Although olfactory testing is widely available, functional OB assessment has been missing. However, recent technical advancements now allow for non-invasive OB assessment through electroencephalographic (EEG) recordings obtained from electrodes placed on the forehead^12^. Importantly, this OB functional measurement has been shown to distinguish PD and ICA patients from healthy controls^12,13^. Longitudinal studies using this approach have the potential to establish early EEG biomarkers that could differentiate between brain-onset and body-onset PD.

Taken together, we believe that a more comprehensive examination of olfactory and OB functions is necessary to advance our understanding of PD pathology and progression.

## Reporting summary

Further information on research design is available in the Nature Portfolio Reporting Summary linked to this article.

## Supplementary information

Reporting summary

## Data Availability

No data are associated with this commentary.

## Code availability

No code is associated with this commentary.
